# Ectopic expression of *MdSPDS1 *in sweet orange (*Citrus sinensis *Osbeck) reduces canker susceptibility: involvement of H_2_O_2 _production and transcriptional alteration

**DOI:** 10.1186/1471-2229-11-55

**Published:** 2011-03-28

**Authors:** Xing-Zheng Fu, Chuan-Wu Chen, Yin Wang, Ji-Hong Liu, Takaya Moriguchi

**Affiliations:** 1 Key Laboratory of Horticultural Plant Biology of Ministry of Education, College of Horticulture and Forestry Sciences, Huazhong Agricultural University, Wuhan 430070, China; 2 National Key Laboratory of Crop Genetic Improvement, College of Horticulture and Forestry Sciences, Huazhong Agricultural University, Wuhan 430070, China; 3 National Institute of Fruit Tree Science, Tsukuba, Ibaraki 305-8605, Japan

## Abstract

**Background:**

Enormous work has shown that polyamines are involved in a variety of physiological processes, but information is scarce on the potential of modifying disease response through genetic transformation of a polyamine biosynthetic gene.

**Results:**

In the present work, an apple *spermidine synthase *gene (*MdSPDS1*) was introduced into sweet orange (*Citrus sinensis *Osbeck 'Anliucheng') via *Agrobacterium*-mediated transformation of embryogenic calluses. Two transgenic lines (TG4 and TG9) varied in the transgene expression and cellular endogenous polyamine contents. Pinprick inoculation demonstrated that the transgenic lines were less susceptible to *Xanthomonas axonopodis *pv. *citri *(Xac), the causal agent of citrus canker, than the wild type plants (WT). In addition, our data showed that upon Xac attack TG9 had significantly higher free spermine (Spm) and polyamine oxidase (PAO) activity when compared with the WT, concurrent with an apparent hypersensitive response and the accumulation of more H_2_O_2_. Pretreatment of TG9 leaves with guazatine acetate, an inhibitor of PAO, repressed PAO activity and reduced H_2_O_2 _accumulation, leading to more conspicuous disease symptoms than the controls when both were challenged with Xac. Moreover, mRNA levels of most of the defense-related genes involved in synthesis of pathogenesis-related protein and jasmonic acid were upregulated in TG9 than in the WT regardless of Xac infection.

**Conclusion:**

Our results demonstrated that overexpression of the *MdSPDS1 *gene prominently lowered the sensitivity of the transgenic plants to canker. This may be, at least partially, correlated with the generation of more H_2_O_2 _due to increased production of polyamines and enhanced PAO-mediated catabolism, triggering hypersensitive response or activation of defense-related genes.

## Background

During the last decade significant progress has been made in citrus production throughout the world. However, world citrus industry is frequently confronted with risk of devastation by a variety of biotic or abiotic stresses. Citrus canker disease, caused by *Xanthomonas axonopodis *pv. *citri *(Xac), is one of the most destructive biotic stresses threatening the citrus production globally [1,2]. The typical symptoms of canker caused by Xac include water-soaked eruptions and pustule-like lesions on leaves, stems and fruits, which can lead to defoliation, dieback and fruit drop, yielding enormous loss of production and fruit quality. Xac can attack a fairly wide spectrum of hosts with variable damage, including most citrus species and some related genera [3]. Although a considerable effort has been tried, to breed a resistant cultivar using traditional breeding methods still remains a big challenge [1,4,5]. Kumquat (*Fortunell *Spp.) has been suggested to be resistant to Xac, however, it is not easy to transfer the resistance from kumquat to citrus via cross hybridization due to a series of natural barriers such as male/female sterility, long juvenile period, high degree of heterozygosity, and polyembryony. At present, the primary strategies for controlling canker disease depend upon an integrated approach including eradication program and use of antibiotics or bactericides [6]. However, it should be pointed out that these strategies are not the ultimate solutions considering the cost, safety to human and animals, consistency and stabilization, and impacts on the environment. Breeding a cultivar resistant to Xac provides the most effective and economical way to control this disease. Genetic engineering paves the way for creating novel germplasms that are otherwise impossible via classic breeding strategy, and has been widely employed to produce disease-resistant materials without greatly altering existing genetic background [7].

Plants have developed mechanisms of physiological, biochemical and molecular responses to protect them against the pathogenic attack, apart from the structural barriers and pre-formed antimicrobial compounds [8-10]. Among these, genetically programmed suicide of the cells at the infection sites, known as hypersensitive response (HR), constitutes an important line of defense against pathogen invasion. Previous studies suggested that presence or accumulation of hydrogen peroxide (H_2_O_2_) played a central role in the orchestration of HR [11,12]. Moreover, H_2_O_2 _serves as a substrate driving the cross-linking of cell wall structural proteins to retard microbial ingress [12]. A great amount of evidences has shown that H_2_O_2 _is also an important molecule to mediate signal transduction in the activation of defense-related genes [12,13]. Therefore, manipulating H_2_O_2 _production to a higher but below the cytotoxic level might be an effective way to battle against the pathogen invasion, leading to enhanced disease tolerance.

The production of H_2_O_2 _in plants undergoing stresses experiences a two-phase process, the rapid and transient phase and the late and persistent phase, but more H_2_O_2 _is generated in the latter phase than in the former one [14-16]. Although the precise role of H_2_O_2 _in each phase remains unclear, H_2_O_2 _produced in the latter phase has been suggested to be closely involved in plant defense response [15]. In addition, in this phase H_2_O_2 _was predominantly produced through the polyamine degradation mediated by either flavine-containing polyamine oxidases (PAO, EC 1.5.3.11) or copper-containing amine oxidases (CuAO, EC 1.4.3.6) [15,17-20]. Polyamines, mainly diamine putrescine (Put), triamine spermidine (Spd) and tetraamine spermine (Spm), are low-molecular-weight natural aliphatic polycations that are ubiquitously distributed in all living organisms. As an important source of H_2_O_2 _production, polyamines have been suggested to be involved in response to pathogen attack or to be responsible for enhanced disease resistance in higher plants [21] based on the following lines of evidence, although the exact mode of action needs to be explicitly clarified. Firstly, the polyamine levels were increased after attack by fungus [22,23], virus [19,24-26] and bacterium [27], implying that polyamine accumulation may be a common event for plant response to various pathogens. Secondly, augmentation of the polyamine level in a host plant through exogenous application of polyamines enhanced resistance to viral or bacterial pathogens [25,27,28]. It is suggested that the endogenous polyamines accumulating under these circumstances may serve as substrates for either PAO or CuAO, leading to production of sufficient H_2_O_2 _that functions in HR or signaling transduction [19,29,30]. This assumption may be plausible as PAO/CuAO-mediated polyamine degradation has been reported to be correlated with the induced tolerance to specific pathogens. For example, inhibition of CuAO activity by an irreversible inhibitor reduced accumulation of H_2_O_2 _and led to a concurrent development of extended necrotic lesions in chickpea when inoculated with *Ascochyta rabiei *[20]. In a recent study, tobacco plants overexpressing a PAO gene yielded more H_2_O_2 _and exhibited preinduced disease tolerance to both bacteria and oomycetes, whereas repression of the PAO by means of using an inhibitor, virus-induced gene silencing or antisense technology suppressed H_2_O_2 _production and then lost HR, coupled with an increase of bacterial growth [30]. All of these findings indicate that accumulation of polyamines and an ensuing degradation play a pivotal role in defense against the pathogens, in particular biotrophic ones [27].

Polyamine biosynthesis in higher plants has been well documented, in which five key biosynthetic enzymes are involved, arginine decarboxylase (EC 4.1.1.19), ornithine decarboxylase (EC 4.1.1.17), *S*-adenosylmethionine decarboxylase (EC 4.1.1.50), Spd synthase (SPDS, EC 2.5.1.16) and Spm synthase (EC 2.5.1.22). As cellular polyamine content can be regulated at the transcriptional level, it is possible to modulate the endogenous polyamine level via overexpression of the polyamine biosynthetic genes, as has been revealed elsewhere [31,32]. It is worth mentioning that although much effort has been invested to elucidate the role of polyamines in disease tolerance, the knowledge is still limited as the data are obtained from only few plant species. The raised question is whether promotion of polyamine biosynthesis/catabolism can be used as an approach to obtain transgenic plants with improved disease resistance in an economically important fruit crop like citrus. Toward understanding this question, we first produced transgenic sweet orange (*Citrus sinensis*) plants overexpressing *MdSPDS1 *isolated from apple [33]. Then we showed that two transgenic lines (TG) with varying mRNA levels of the transgene were less susceptible to Xac than the wild type plants (WT), which might be correlated with production of H_2_O_2 _and/or up-regulation of transcription levels of defense-related genes. To our knowledge, this is the first report on improving disease resistance in a perennial fruit crop *via *transformation of a gene involved in polyamine biosynthesis, adding new insight into the functions of polyamines for engineering biotic stress tolerance.

## Results

### Transformation and regeneration of plants from embryogenic calluses

**Figure 1:**
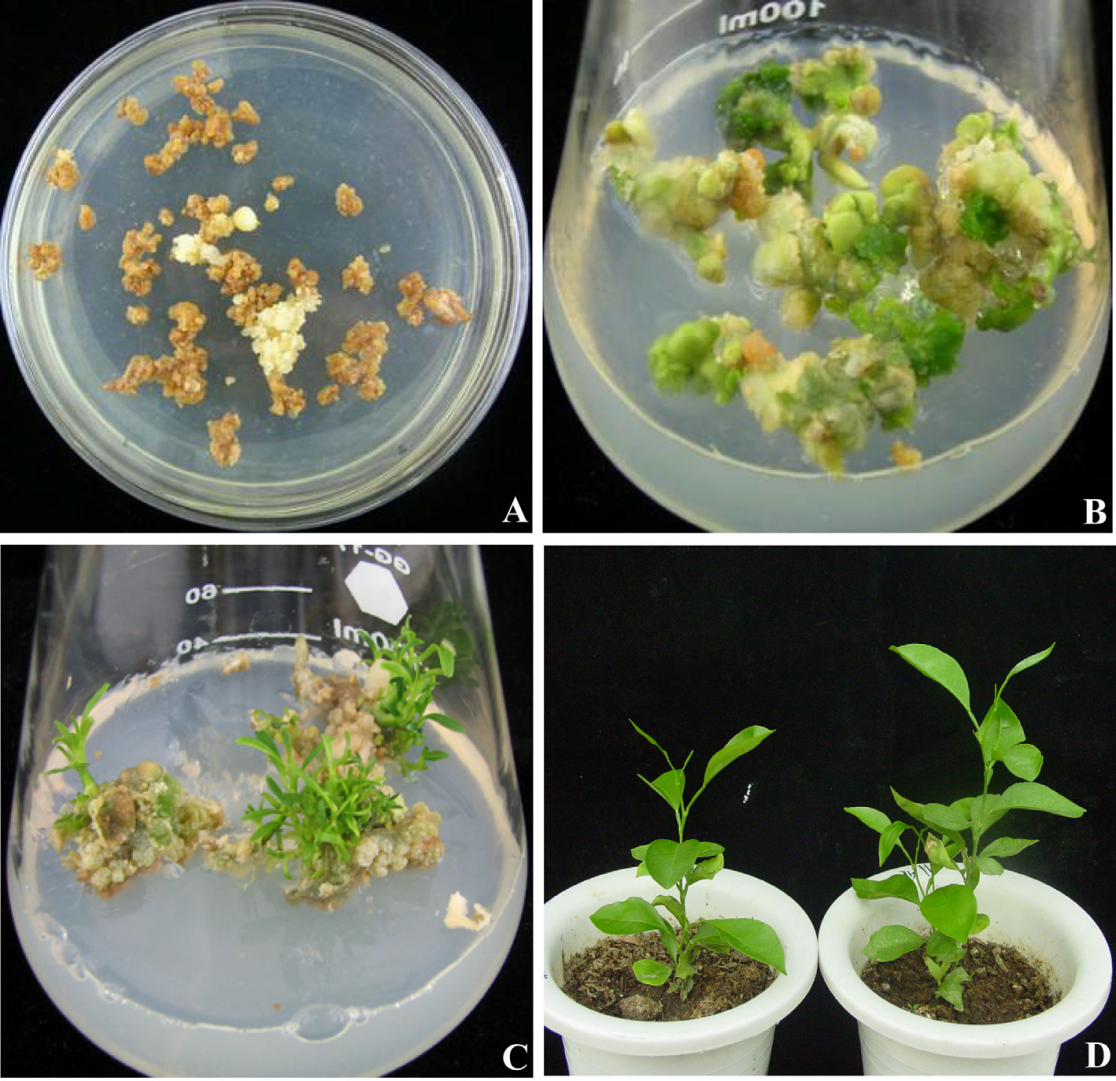
**Regeneration of transgenic plants from 'Anliucheng' embryogenic callus infected with *Agrobaterium tumefaciens *containing *MdSPDS1 *gene**. (A) Selection of the callus on kanamycin-containing medium. (B) Induction of embryoids from the callus that survived after several rounds of selection. (C) Regeneration of multiple shoots from cotyledonary embryoids. (D) Wild type (left) and a transgenic line (TG9, right) grown in a soil pot.

To obtain transgenic plants, the embryogenic calluses of 'Anliucheng' sweet orange were infected with the *Agrobacterium tumefaciens *strain LBA4404 containing pBI121::*MdSPDS1 *and a neomycin phosphotransferase gene (*NPTII*). On the selection medium containing kanamycin, most of the infected calluses turned brown within 1 month, while the kanamycin-resistant calluses were still white (Figure 1A). The kanamycin-resistant calluses were then cultured on the fresh selection medium for further selection and multiplication. At last, the surviving calluses after several rounds of selection were transferred to embryoid-inducing medium to induce embryogenesis (Figure 1B). Thereafter, mature cotyledonary embryoids were cultured on the shoot-inducing medium to regenerate shoots (Figure 1C). When the shoots were 1.5 cm in length, they were excised and moved to root-inducing medium to get rooting plantlets. Two months after rooting, the plantlets were planted in the soil pots and kept in a growth chamber for further growth (Figure 1D).

### Molecular confirmation of the regenerated plants

**Figure 2:**
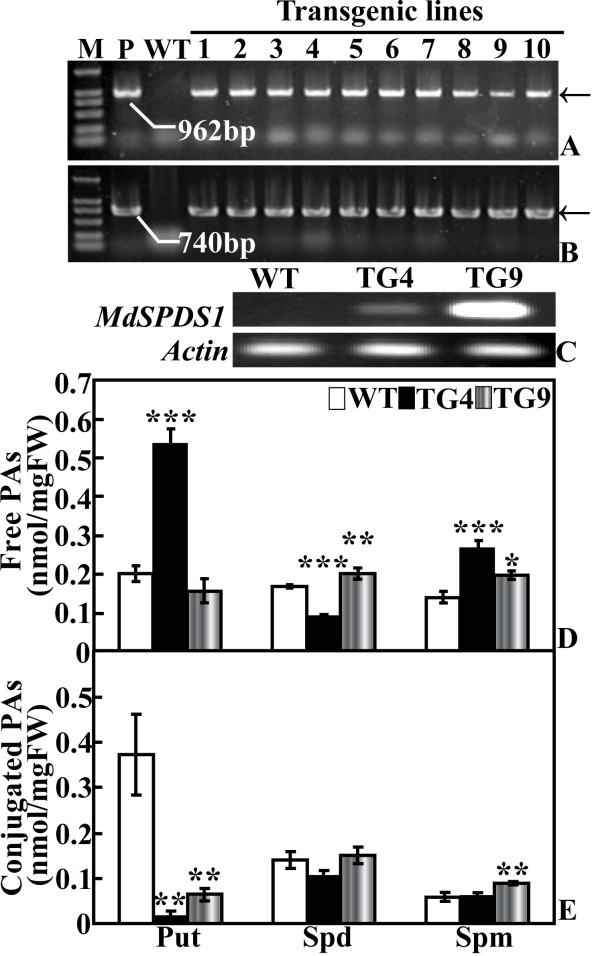
**Molecular analysis and polyamine content of the transgenic plants**. PCR amplification of transgenic lines that are transferred to soil pots via specific primers of *CaMV35S-MdSPDS1 *(A) and *NPTII *(B). (C) Semi-quantitative RT-PCR analysis on the expression level of *MdSPDS1 *in the wild type (WT) and two transgenic lines (TG4 and TG9). (D-E) Analysis of free (D) and conjugated (E) polyamine content by HPLC in fully expanded leaves sampled from the WT and transgenic plants grown under the same conditions. *, ** and *** indicate the values are significantly different compared with WT at significance level of *P *< 0.05, *P *< 0.01 and *P *< 0.001, respectively.

PCR using genomic DNA as template was performed to verify the integration of *MdSPDS1 *in the regenerated plants. The amplification with specific primers showed that expected fragments with the same size as that of the plasmid were produced in all of the ten tested lines, but not in the WT (Figure 2A-B), indicating that they were putative transformants. Overexpression of the *MdSPDS1 *gene was further analyzed in two lines (TG4 and TG9) by semi-quantitative RT-PCR. mRNA levels of *MdSPDS1 *were detected in both TG4 and TG9, but the level is higher in the latter line (Figure 2C).

### Free and conjugated polyamine levels in the transgenic lines and WT under normal conditions

Free polyamine levels of TG4, TG9 and WT were determined with HPLC (Figure 2D). As compared with the WT, TG4 had significantly higher level of Put (538.9 vs. 201.7 nmol/g FW), while Put of TG9 (156.0 nmol/g FW) was slightly reduced. Spd levels of TG4 (87.4 nmol/g FW) and TG9 (199.2 nmol/g FW) were significantly reduced and increased, respectively, in comparison to the WT (167.8 nmol/g FW). Spm content in both lines (268.0 nmol/g FW for TG4, 197.3 nmol/g FW for TG9) were significantly increased relative to the WT (136.7 nmol/g FW). Conjugated Put levels of TG4 and TG9 were significantly reduced compared with the WT, and the largest decrease was detected in TG4 (Figure 2E). The conjugated Spd of TG4 was slightly but insignificantly lower than the WT and TG9 that were close to each other, while the conjugated Spm level of TG9 was significantly higher than that of WT and TG4.

### Xac challenge of the transgenic plants and the WT

**Figure 3:**
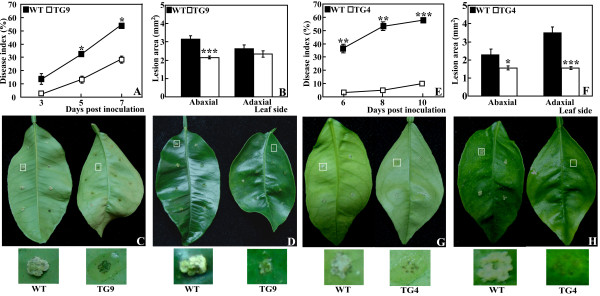
**Canker disease tolerance assay of the wild type (WT) and the transgenic lines (TG4 and TG9)**. Disease index (A, E) and lesion area (B, F) of WT, TG9 (A-D) and TG4 (E-H) after inoculation with Xac. Comparison between TG9 and WT, TG4 and WT was done in different inoculation experiment. Asterisks show that the values are significantly different compared with the control (* for *P *< 0.05, ** for *P *< 0.01 and *** for *P *< 0.001). Representative photographs showing symptoms on the abaxial (C, G) and adaxial (D, H) sides of the leaves from WT/TG9 (C-D) and WT/TG4 (G-H). Selected inoculation sites of the leaves were zoomed in and shown below the corresponding photos.

The accumulation of Spd and Spm, especially Spm, led us to test the defense capacity of the transgenic plants against the Xac pathogen as Spm has been shown to be an endogenous inducer for defense-related genes [25,34]. To this end, TG9 and the WT were challenged with Xac by pinprick inoculation under the same conditions, followed by comparison of timing of canker symptom, disease index (DI) and lesion size between them. DI of WT at 3, 5 and 7 days post inoculation (DPI) was 13.21, 32.14 and 54.64, about 6.17, 2.43 and 1.91 times larger than that of TG9, respectively (Figure 3A). On 5 DPI, large white spongy pustules were formed at the inoculation sites in both abaxial and adaxial sides of the WT leaves, whereas TG9 showed the symptom only at fewer inoculation sites of the adaxial side (Figure 3C-D). Although white spongy pustules could be detected in both the WT and TG9 at 7 DPI, size of the lesions in the WT was about 1.5 times bigger than that of TG9 on the abaxial side (3.15 mm^2 ^for WT and 2.15 mm^2 ^for TG9). Similarly, on the adaxial side, the WT had bigger lesions (2.65 mm^2^) than TG9 (2.34 mm^2^, Figure 3B). Inoculation of TG4 and the WT in a different set of experiments also showed that TG4 was also less susceptible to citrus canker (Figure 3E-H), although the timing of canker occurrence varied from that of TG9. These data indicate that both TG9 and TG4 were more tolerant to canker disease than the WT. To dissect the potential mechanisms underlying the enhanced canker tolerance, we performed in-depth work using TG9 as it had higher expression level of *MdSPDS1 *and Spd and Spm level.

### TG9 accumulated more H_2_O_2 _than the WT after Xac inoculation

**Figure 4:**
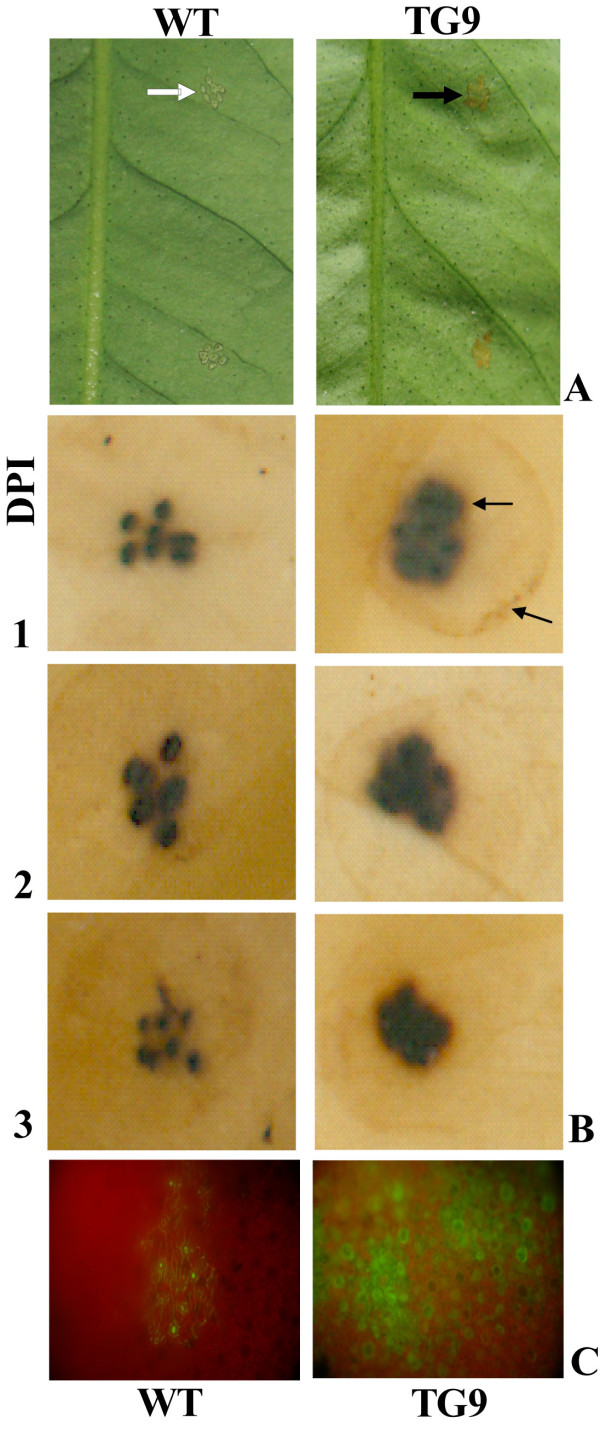
**Hypersensitive reaction and assay of H_2_O_2 _at the inoculation sites and in the neighboring regions of the wild type (WT) and transgenic line (TG9) leaves after Xac inoculation**. (A) Representative photos showing the WT and TG9 leaves after 3 d of Xac inoculation. Arrows show the occurrence of HR at the inoculation sites. (B-C) *In situ *accumulation of H_2_O_2 _in the WT and TG9 leaves, as revealed by histochemical staining assay via 3, 3'-diaminobenzidine (B) and H_2_DCF-DA (C), respectively.

It is noted necrosis was observed at the inoculation sites of TG9 leaves when they were inoculated with Xac, a sign of HR, which was otherwise absent in the WT (Figure 4A), implying that the transgenic plant might experience rapid cell death upon Xac infection. As H_2_O_2 _plays an essential role in the orchestration of HR, accumulation of H_2_O_2 _at the infection sites and in the neighboring regions was visually detected by DAB and H_2_DCF-DA, respectively. At 1 DPI of Xac inoculation, both TG9 and the WT had brown spots at the infected sites. However, compared with the WT, TG9 showed deeper brown color than the WT. Interestingly, a brown circle was viewed around the infected sites of TG9, which was not detected in the WT (Figure 4B). A similar staining pattern was noticed at 2 and 3 DPI, suggesting that TG9 might accumulate higher H_2_O_2 _at the infection sites than the WT.

Since DAB staining was difficult to reveal the H_2_O_2 _accumulation in the regions near the inoculation sites, H_2_DCF-DA staining was used to determine H_2_O_2 _therein using the samples collected at 2 DPI. As can be seen in Figure 4C, TG9 leaves showed more abundant green fluorescence than the WT, indicating presence of higher H_2_O_2 _level in TG9 than in the WT.

### TG9 had higher PAO, SOD and CAT activity than the WT after Xac attack

**Figure 5:**
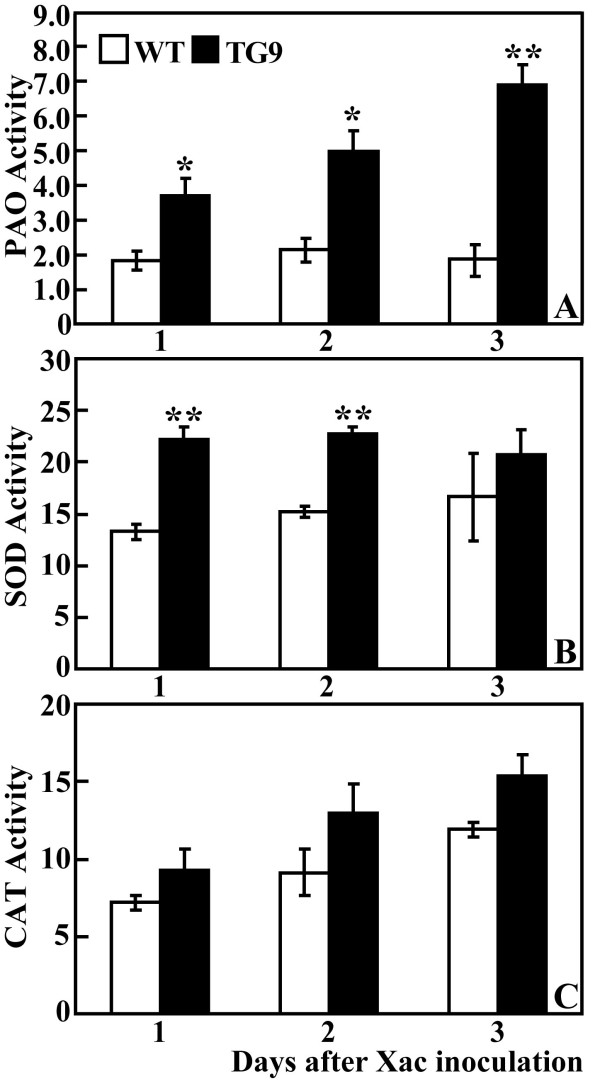
**Analysis of PAO, SOD and CAT activities after Xac infection**. PAO activity (nmol acetylspermine/min/mg protein, A), SOD activity (U/mg protein, B) and CAT activity (U/mg protein, C) were analyzed in the WT and TG9 leaves sampled on 1, 2 and 3 DPI. * and ** indicate the values are significantly different compared with WT at significance level of *P *< 0.05 and *P *< 0.01, respectively.

PAO-mediated polyamine degradation is an important pathway for H_2_O_2 _production, efforts were thus made to investigate PAO enzyme activity in the WT and TG9 leaves sampled at 1, 2 and 3 DPI. Measurement showed that PAO activity of the WT did not vary greatly despite a negligible increase at 2 DPI, while that of TG9 was enhanced over inoculation time. As a result, PAO activity of TG9 was significantly higher than that of the WT at the three time points (Figure 5A).

Antioxidant enzymes have been shown to be important for homeostasis of ROS, so we also examined activities of two enzymes involved in H_2_O_2 _production and scavenging, superoxide dismutase (SOD) and catalase (CAT), in the WT and TG9 at 1, 2 and 3 DPI. SOD activity exhibited minor change upon Xac infection, but it was higher in TG9 compared with the WT, particularly at 1 and 2 DPI (Figure 5B). Xac inoculation induced a progressive increase of the CAT activity in both TG9 and the WT. However, they were statistically insignificantly different from each other at any time point (Figure 5C).

### Changes of free polyamines after the Xac infection

**Figure 6:**
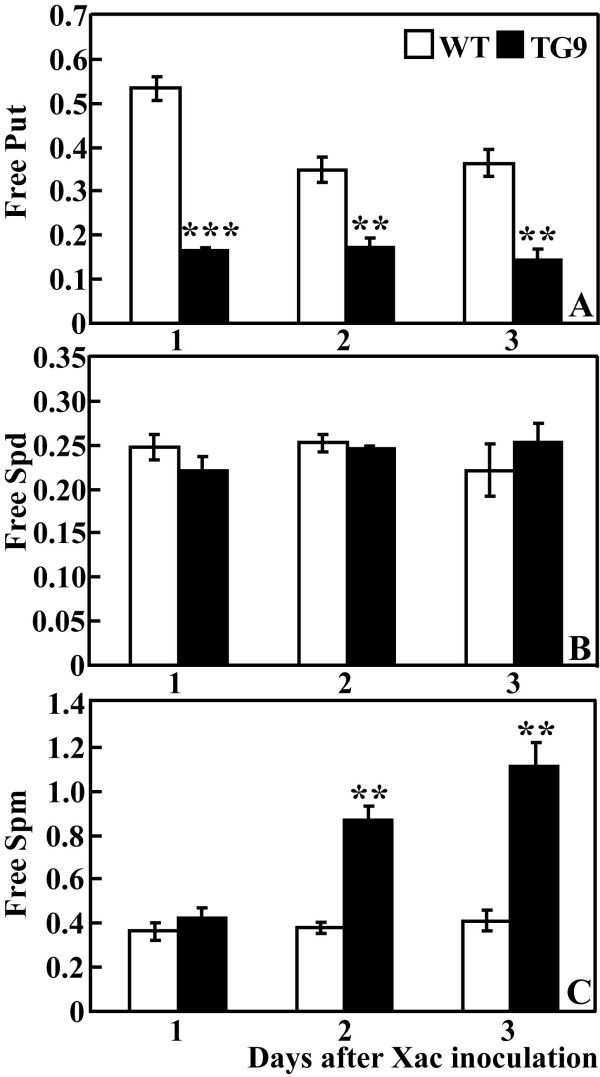
**Analysis of free polyamine contents in the wild type (WT) and transgenic line (TG9) after Xac infection**. Free putrescine (A), spermidine (B) and spermine (C) contents (nmol/mg FW) were analyzed in the WT and TG9 leaves sampled on 1, 2 and 3 DPI. ** and *** indicate the values are significantly different compared with WT at significance level of *P *< 0.01 and *P *< 0.001, respectively.

Free polyamine levels were also evaluated after the Xac infection in the present study. Xac attack reduced free Put level in the WT, whereas TG9 underwent slight change and the Put content in TG9 was still significantly lower than that of the WT at any time point (Figure 6A). Free Spd in the WT and TG9 was similar and showed slight alterations during the period (Figure 6B). At 1 DPI, no differences in free Spm level were observed between TG9 and the WT. Although WT exhibited no change at 2 and 3 DPI, the Spm in TG9 presented an increase, leading to significantly higher levels in TG9 relative to WT at the last two time points (Figure 6C).

### Treatment with a PAO inhibitor enhanced Xac susceptibility

**Figure 7:**
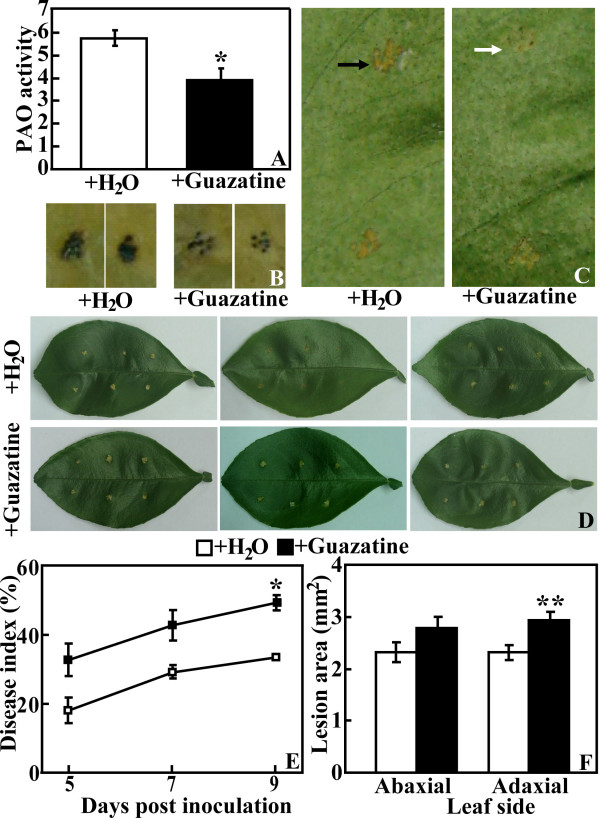
**Effect of PAO inhibitor, guazatine acetate (Guazatine), on canker disease susceptibility of the transgenic line (TG9)**. (A-C) PAO enzyme activity (nmol acetylspermine/min/mg protein, A), DAB staining (B) and hypersensitive response (C, shown by arrows) of leaves treated with Guazatine or water (H_2_O), collected on 3 DPI. (D) Representative photographs showing symptoms of Guazatine or H_2_O-treated leaves after Xac inoculation for 9 d. (E-F) Disease index (E) and lesion size (data of 9 DPI, F) of the leaves treated with Guazatine or H_2_O after Xac infection. * and ** indicate the values are significantly different compared with WT at significance level of *P *< 0.05 and *P *< 0.01, respectively.

The above data showed that PAO activity in TG9 was increased after Xac infection, consistent with the accumulation of H_2_O_2_. In order to know if the PAO-mediated H_2_O_2 _production was responsible for the canker tolerance, a PAO inhibitor (guazatine acetate) was used to treat TG9 before Xac inoculation. In a preliminary experiment, we showed that the inhibitor did not arrest the growth of Xac bacteria (data not shown). When the leaves were treated with the inhibitor, PAO activity was reduced by 32.2% at 3 DPI (Figure 7A). Interestingly, at this time H_2_O_2 _production of the inhibitor-treated samples was lower than that treated with water (Figure 7B). In contrast, HR was more conspicuous at the inoculation sites of the leaves without inhibitor pretreatment (Figure 7C). Moreover, the inhibitor-treated leaves exhibited more serious canker symptom over a 9-d inoculation experiment when compared with the water-treated ones, as manifested by the higher DI (Figure 7D-E) and larger lesion size on the abaxial and adaxial sides (Figure 7F). All of these data suggested that repression of PAO by the inhibitor resulted in production of less H_2_O_2 _and a concomitant increase of sensitivity to Xac attack.

### Expression analysis of defense-related genes before and after the Xac inoculation

**Figure 8:**
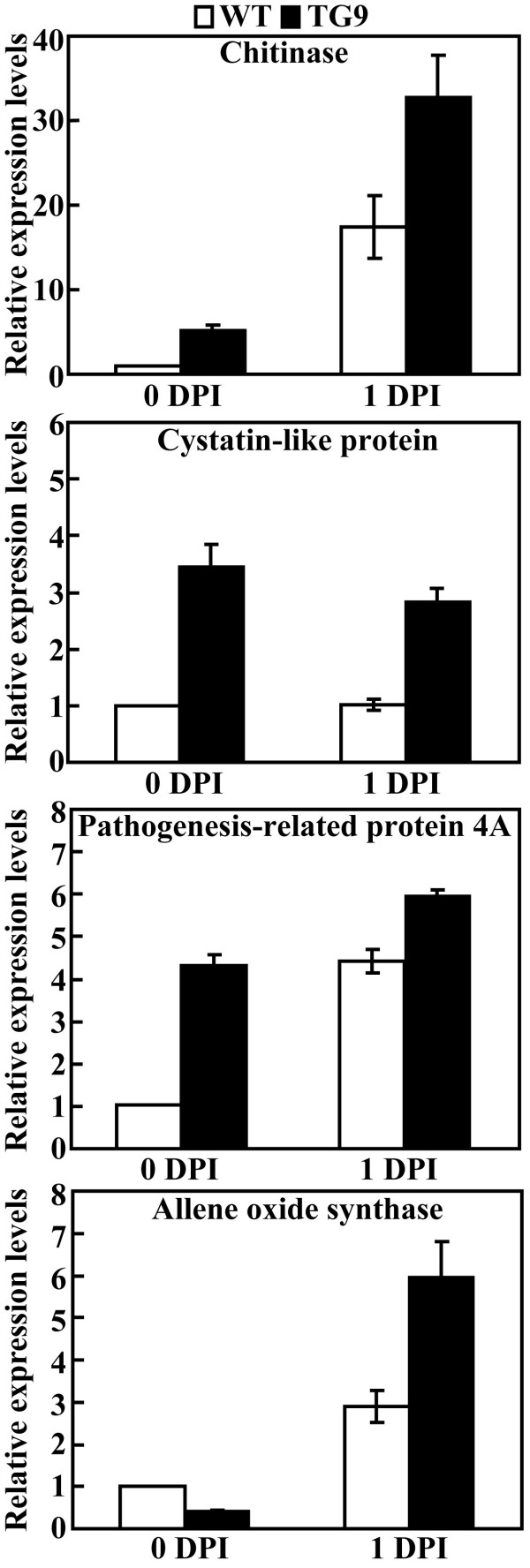
**Quantitative real-time RT-PCR analysis on expression levels of defense-related genes in the wild type (WT) and transgenic line (TG9) before and after Xac inoculation**. Transcriptional levels of chitinase, cystatin-like protein, pathogenesis-related protein 4A (PR4A) and allene oxide synthase (AOS) were assessed by quantitative real-time RT-PCR in the WT and TG9 before (0 DPI) and 1 d after (1 DPI) Xac inoculation.

Disease resistance is a complex process in which many defense-related genes are activated to play crucial roles. To elucidate whether or not mRNA levels of defense-related genes are influenced in TG9 compared with the WT, transcript levels of genes encoding chitinase, cystatin-like protein, pathogenesis-related (PR) protein 4A (PR4A) and allene oxide synthase (AOS) were assessed by real-time quantitative RT-PCR. As shown in Figure 8, relative expression levels of all the tested genes were prominently enhanced in TG9 relative to the WT before or after Xac inoculation, except AOS gene at 0 DPI. These data suggest that the defense-related genes were constitutively activated in the transgenic plant.

## Discussion

Citrus canker is a devastating disease afflicting citrus production worldwide. In order to create novel germplasms with reduced susceptibility to canker, genetic transformation of antibacterial peptides or *R*-genes has been tried before this work. For instance, Barbosa-Mendes et al. [35] introduced a gene encoding harpin protein into 'Hamlin' sweet orange and the resultant transgenic lines showed reduction in Xac susceptibility. Very recently, Mendes et al. [36] reported that transformation of rice *Xa21 *gene into sweet orange gave rise to enhanced tolerance to canker. Herein, we show that a polyamine biosynthetic gene is successfully introduced into sweet orange and the transgenic plants are less susceptible to citrus canker, which opens a new avenue for producing novel citrus germplasms resistant to a biotic stress. Despite the fact that genetic transformation of polyamine biosynthetic genes has been shown to confer abiotic stress tolerance [31,32,37,38] information is relatively scarce concerning application of this strategy to the biotic stress engineering. So far, only polyamine catabolic genes have been engineered to enhance resistance to pathogen challenge [20,30,39]. Our work gains new insight into new function of the genes involved in polyamine biosynthesis.

Although *MdSPDS1 *was overexpressed in TG4 and TG9, the endogenous polyamine levels in these two lines differed from each other. The difference may be plausible since TG4 and TG9 arose from independent transformation events, suggesting polyamine biosynthesis might be variably modulated in the transgenic plants expressing the same gene. It is worth mentioning that variation of polyamine levels in the genetic transformants overexpressing polyamine biosynthetic genes has been reported in earlier studies [32,40]. Our data and the data of earlier work demonstrate that there is a complex regulation of intracellular polyamine contents under these circumstances, which may vary among plant species, transgene type and physiological conditions. A striking finding herein is the extremely high level of free Put level in TG4 relative to the WT and TG9. It has been documented that endogenous cellular polyamine level is dependent upon several interconnected pathways, such as *de novo *synthesis, degradation and conjugation, but the exact contribution of an individual pathway is not yet identified. In TG4, high level of free Put was largely consistent with the low level of its conjugated counterpart, implying that in this line the conjugated Put might have been enormously converted to free part. This sounds reasonable as the conjugated polyamines are of particular importance for the regulation of intracellular polyamine levels [41]. However, this scenario does not hold true for Put level of TG9 and Spd/Spm level of both transgenic lines as the interrelationship between free and conjugated form was not established, indicating that relative proportion of the free and conjugated polyamines is diversified among different plants [41]. On the other hand, the possibility of back conversion from Spd to Put in TG4 might also partially explain the high Put level (also lower Spd) in this line. Although we could not present evidence to support this presumption herein, such conversion has been previously reported in other plants [42,43]. As for TG9, despite a substantial increase of the *MdSPDS1 *mRNA, endogenous Spd and Spm levels were just slightly increased, which demonstrated that no direct correlation exists between the transcription level of a biosynthetic gene and the product of the protein activity [37,44]. In previous studies overexpression of the polyamine biosynthetic genes like *SAMDC *or *SPDS *has also been shown to bring about very limited accumulation of Spd and/or Spm, which may be ascribed to tight homeostatic regulation of these compounds at cellular level [32,40]. In addition, TG9 has lower level of free Put compared with the WT despite presence of higher expression of the transgene. At this stage it is still ambiguous to unravel an exact reason for the observed phenomenon as the polyamine biosynthetic control is invested at multiple interdependent steps [44]. One possibility is the timely conversion into the downstream compounds (Spd) by SPDS due to overexpression of the gene, as evidenced by the slightly higher Spd. However, other possibilities, such as repressed synthesis or stimulated degradation, could not be fully ruled out.

HR was observed at the inoculation sites in TG9, whereas it was largely absent in the WT (Figure 4A). Plants possess an innate immune system to defend themselves against the pathogens, and HR serves as an important protective strategy to limit pathogen spread through suicidal death of the host cells [10]. Interestingly, the induction of HR is concomitant with accumulation of higher H_2_O_2 _in TG9 when compared with the WT. It is known that activation of HR is relevant to the abundant production of reactive oxygen species (ROS), also referred to as oxidative burst, in which H_2_O_2 _plays a significant part [15]. Therefore, it seems likely that TG9 accumulated more H_2_O_2 _than the WT, which effectively triggered the cell death at the inoculation sites, leading to an enhancement of canker disease resistance. DAB staining of the inoculated sites supported this likelihood. The question then arises as to how TG9 produced more H_2_O_2 _than WT. As mentioned earlier, H_2_O_2 _generated by polyamine degradation plays an important role in plant defense response upon the pathogen invasion [15,18-23]. This scenario led us to focus our research efforts on the polyamine degradation via PAO as this process has been suggested to be an important source of H_2_O_2 _production during pathogen infection [21]. It can be seen in Figure 5A that after Xac inoculation TG9 showed continuous increase in the PAO activity, significantly higher than the WT at 1, 2 and 3 DPI. Our results support previous studies in which PAO activity was induced upon exposure to pathogen challenge [15,22,23]. Presence of the higher PAO activity in TG9 agrees well with the accumulation of more copious H_2_O_2_, indicating that PAO-mediated polyamine oxidation might contribute to the accumulation of H_2_O_2 _after Xac infection, which was further supported by the application of the PAO inhibitor. In addition, it is noticed that use of the inhibitor alleviated HR, coupled with more prominent canker symptom, implying that PAO-mediated polyamine oxidation, producing H_2_O_2 _that triggers hypersensitive cell death, is involved in Xac tolerance in the transgenic line.

However, the interpretation of these results should be treated cautiously at this stage as other possibility of H_2_O_2 _production could not be exclusively precluded, at least via two other pathways. First, we still could not rule out the possibility of involvement of CuAO in mediating the polyamine oxidation to produce H_2_O_2 _after the Xac attack. Although in the present study we had no data to elucidate the function of CuAO in defense response to Xac, this enzyme and its activity have been previously shown to be essential for the H_2_O_2 _production in protection against pathogens [20]. Second, H_2_O_2 _accumulation might be also relevant to the antioxidant system, particularly SOD that catalyzes the conversion of superoxide anion (O_2_^-^) into H_2_O_2_. In our study, TG9 had higher SOD activity compared with the WT, consistent with earlier work that endogenous SOD activity was promoted when cellular polyamine contents increased [45,46]. It is conceivable that regardless of the transgenics, both the WT and TG9 might first accumulate O_2_^- ^when exposed to Xac. As TG9 had higher SOD activity, the O_2_^- ^produced in this line might be dismutated to generate H_2_O_2 _in a more efficient manner. As the CAT activity was similar between WT and TG9 (no statistical difference here), the H_2_O_2 _in these two lines may be equivalently removed by CAT. Since TG9 had a better supply of H_2_O_2 _by the higher SOD activity the outcome is that it accumulated more H_2_O_2_, which was controlled by CAT below the destructive concentration and in the meantime can function well in modulating the stress response.

After the Xac inoculation, free Put of TG9 was still lower than that of the WT throughout the experimentation. Papadakis and Roubelakis-Angelakis [47] have proposed that high concentration of Put prevented cell death, which suggested that the lower Put level in TG9 may create a favorable situation stimulating hypersensitive cell death. On the contrary, although TG9 and the WT exhibited no difference in free Spd content, the former contained higher Spm than the latter, particularly at the last two time points. Induction of polyamines agrees with previous results in which various biotic stresses caused an increase of cellular polyamines [18,19]. An interesting finding in our work is that Spm was accumulated along with higher PAO activity in TG9 relative to the WT. It seems tempting to suggest that upon the Xac infection Spm was simultaneously synthesized and degraded, consistent with the accumulation of H_2_O_2 _mentioned above. This phenomenon has also been reported in an earlier study on tobacco treated with an elicitor derived from *Phytophthora cryptogea *[15]. Our data and those of others indicated that the polyamine synthesis is stimulated in plants upon pathogen attack, providing enough substrate pool, which sequentially initiates its exodus to the apoplast and triggers the polyamine catabolism [43,48]. This result also demonstrates that the PAO-mediated catabolism does not cause a concurrent reduction of the corresponding polyamine, which may be ascribed to the fact that there is a feedback stimulation of the polyamine synthesis by the activated catabolism or that only a small fraction of free polyamine (Spm) is allocated for the catabolic branch.

Spm has been proposed as a signaling molecule that can induce cellular signal transduction pathway [25,31,34,49]. Apart from local HR, H_2_O_2 _serves as a diffusible signal to activate defense genes in the adjacent cells [12]. In this work, TG9 had higher Spm and harbored more H_2_O_2 _after attack by the Xac, which compelled us to investigate expression of several defense-related genes before or after Xac inoculation so as to gain a further insight into the molecular mechanism underlying the disease response. The transcript levels of the tested genes were higher in TG9 than in the WT before Xac infection, except the *AOS *gene. Upon Xac challenge, all genes were induced to more intense extent in TG9 than in the WT, but the magnitude of induction varied among the genes. Chitinase (PR3), cystatin-like protein (PR6) and PR4A (PR4) are PR proteins responsible for systemic acquired resistance [50]. PR proteins have been shown to be induced by exogenously applied Spm and by H_2_O_2 _[25], suggesting that TG9 might synthesize more abundant PR proteins to protect it against the pathogen infection, even in the absence of a biotic stress shock. AOS is a cytochrome P450 (CYP74A) that catalyzes the first committed step in the biosynthesis of jasmonic acid (JA). JA has been suggested to play a pivotal role in the defense response and the mediation of induced systemic resistance [51,52]. Induction of this gene after Xac infection implies that JA synthesis may be involved in the resistance to Xac. Stronger activation of these stress-related genes in TG9, particularly after Xac infection, indicates that the transgenic plant might deploy more robust defense machinery against the invading pathogen (Xac herein). Our present finding also demonstrates that an extensive transcriptional modification has taken place due to the ectopic expression of a polyamine biosynthetic gene, leading to a build-up of disease protection mechanisms in the transgenic plants, although the exact mode of action remains to be clarified.

Taken together, overexpression of *MdSPDS1 *increased the endogenous cellular Spm, which may serve as a signal to directly trigger expression of the defense-related genes under normal conditions. Upon the Xac attack, Spm synthesis was elevated in the transgenic line and PAO was accordingly more prominently activated through an as yet unidentified mechanism, generating a higher level of H_2_O_2 _that plays dual roles in either evoking the hypersensitive cell death or activating expression of the defense-related genes, which may function in concert or independently to reduce canker susceptibility. It has to be pointed out that production of more H_2_O_2 _might also contribute to the cell wall reinforcement or directly act as a microbicidal compound during the pathogen invasion. Although we could not decipher the definite mechanism of action regarding H_2_O_2 _herein, our work, together with earlier ones using PAO gene engineering [27,30,43,48], demonstrates that polyamine synthesis and catabolism is an important player for modifying plant responses to diseases, which offers a new approach for engineering plant disease tolerance.

## Conclusion

Transgenic sweet orange plants over-expressing *MdSPDS1 *have been successfully regenerated, which are less susceptible to the canker disease caused by Xac as compared with the untransformed plants.

Using the transgenic line (TG9) with the highest transgene expression level we demonstrated that the reduced Xac susceptibility might be ascribed to accumulation of H_2_O_2 _which was, at least partially, produced by PAO-mediated polyamine catabolism. In addition, genes involved in disease defense were up-regulated to larger extent in the transgenic line relative to the untransformed plant.

To our knowledge, this is the first report on enhancing biotic stress tolerance via genetic manipulation of a polyamine biosynthetic gene in an economically important perennial crop.

## Methods

### Plant materials, transformation and regeneration

The embryogenic calluses of 'Anliucheng' sweet orange (*Citrus sinensis *Osbeck) were subcultured once a month on the callus growth medium containing salts of MT [53], 7.8 g/l agar, and 35 g/l sucrose (pH 5.8). After 3 cycles of subculture, the calluses were used for *Agrobacterium tumefaciens*-mediated genetic transformation. The *A. tumefaciens *strain LBA4404 carries pBI121 vector with the *MdSPDS1 *gene and neomycin phosphotransferase II (*NPTII*) gene under the control of a *CaMV*35S promoter. The transformation, selection and regeneration were carried out as previously described [54]. The rooting plants were transferred to soil pots and cultured in a growth chamber.

### Molecular confirmation of the transformants

Genomic DNA was extracted according to Cheng et al. [55]. To confirm the transgenic plants, PCR amplifications were performed with two pairs of primers, one for *NPTII *gene (forward, 5'-AGACAATCGGCTGCTCTGAT-3'; reverse, 5'-TCATTTCGAACCCCAGAGTC-3') and the other for *CaMV*35S and *MdSPDS1 *(forward, 5'-GATGTGATATCTCCACTGACGTAAG-3'; reverse, 5'-ACGAAGAGCATTAGC TACTGTC-3'). Each PCR reaction was composed of 100 ng DNA, 1 × reaction buffer, 2 mM MgCl_2_, 0.2 mM dNTP, 0.5 U of DNA polymerase (*Taq*, Fermentas) and 0.4 μM of each primer in a total volume of 20 μl. PCR amplification was performed at 94°C for 5 min, followed by 35 cycles of 94°C for 45 s, 56°C for 45 s, 72°C for 1 min (for *NPTII*) or 1.5 min (for *CaMV*35S and *MdSPDS1*), and a 5-min extension at 72°C. The PCR products were electrophoresesed on 1.0% (w/v) agarose gel containing 0.5 μg/ml ethidium bromide and visualized under UV transillumination.

Semi-quantitative RT-PCR was employed to examine overexpression of the transgene. For this purpose, total RNA was isolated from the WT and two PCR-confirmed transgenic plants according to Liu et al. [56]. Each RNA sample was treated with PCR amplification-grade DNaseI (Takara, Dalian, China) at 37°C to exclude DNA contamination. One μg of the total RNA was used for cDNA synthesis using the ReverTra Ace-α-™ kit (Toyobo, Japan) following the manufacturer's instructions. RT-PCR using the cDNA template in each reaction and a pair of specific primers for *MdSPDS1 *(forward, 5'-GGAGCCTGATTCTGTCTCCGCTG-3'; reverse, 5'-CCTTTCCATATGTCGCTGA CTGG-3') was carried out with 28 cycles of 40 s at 94°C, 40 s at 56°C and 40 s at 72°C. As an internal positive control, the same cDNA was also amplified with a pair of *Actin *primers (Table 1, [57]) using the procedure mentioned above.

**Table 1:** Primers pair used for Real-time quantitative RT-PCR

*Genes*	*Primers (5'-3')*		*Accession* * numbers*	*Size of* * amplicon*
	Forward	Reverse		
Chitinase	TCTTGCCCTAGCTTTTCCCAC	GCAATCTCACGCTTCGAAACTT	AF090336	204 bp
Cystatin-like protein	GACCCCAAGGAGAAGCACGT	CCCTTCTCCACGCTCTCGAA	AF283536	101 bp
AOS	ATTCCACCTACACGGAGGCAT	TAACGGAGCGAGCTGAAACAG	AY243478	203 bp
PR4A	GGAGGCTTAGATTTGGACGAAGG	ACATAACTGTAGTGCCCATGAGC	CB250274	260 bp
Actin	CATCCCTCAGCACCTTCC	CCAACCTTAGCACTTCTCC	BQ623464	190 bp

### Quantification of free and conjugated polyamines by high-performance liquid chromatography (HPLC)

A previous method described by Liu and Moriguchi [58] was used to extract free polyamines. For this purpose, fully expanded leaves were sampled from young flushes of the WT and transgenic plants grown at the same time. About 0.1 g of the leaf tissues was homogenized in 1 ml of 5% cold perchloric acid (PCA) for 30 min on ice. After centrifugation at 12000 rpm (4°C) for 15 min the supernatant was shifted to a new tube. One ml of 5% PCA was added to the pellet and kept on ice for 30 min before centrifugation under the same conditions. The supernatant from two rounds of centrifugation was mixed, and 500 μl of it was benzoylated according to Liu et al. [59]. The supernatant was mixed with 10 μl of benzoyl chloride and 1 ml of 2M NaOH, which was vortexed for 30 sec and then incubated for 25 min in water bath under 37°C. Thereafter, the benzoylation of polyamines was leached with 2 ml of ethyl ether, vacuum dried in a concentrator (Eppendorf 5301, Germany) and re-dissolved with 100 μl of methanol (HPLC grade). The benzoyl-polyamines (20 μl) were analyzed using an Agilent HPLC system (USA) equipped with a C_18 _reversed phase column (4.6 mm × 150 mm, particle size 5 μm) and a UV-detector according to Shi et al. [46] with minor modification. Preparation of the conjugated polyamines was performed as described by Liu et al. [60] with minor modification. An aliquot of the above-mentioned supernatant (500 μl) was mixed with an equivalent volume of 12 M HCl for 18 h at 110°C. After the acid hydrolysis, HCl was evaporated by heating at 80°C. The resulting residues were re-suspended in 100 μl of 5% PCA, followed by benzoylation and measurement as mentioned above. Quantification of the polyamines was done in triplicate.

### Pinprick inoculation of the leaves with Xac

*Xanthomonas axonopodis *pv. *citri *(Xac), a kind gift by Prof. Ni Hong (Huazhong Agricultural University, Wuhan, China), was shaken overnight (200 rpm) in liquid medium (sucrose 20 g/l, peptone 5 g/l, MgSO_4 _0.25 g/l, K_2_HPO_4 _0.5 g/l, pH 7.2) at 28°C, which were collected by centrifugation and re-suspended in sterile tap water at a concentration of 10^8 ^cells/ml before inoculation. The leaves collected from new flushes of the transgenic lines and the WT were washed with distilled water and then subjected to inoculation on the abaxial side using an insect pin (0.2 mm in diameter). Four inoculation sites of 1.5 mm^2 ^in area (each is composed of 5-7 pricks) were made on each side of the midvein. An aliquot of 10 μl of the bacterial suspension (Xac) was dropped onto each inoculation site. Thereafter, the leaves were placed above wet filter paper in Petri dishes, which were sealed with parafilm to maintain high humidity conducive for bacterial growth in the leaves. The Petri dishes were kept in dark at 28°C in a plant growth chamber, and pictures were taken at 0, 3, 5, 7 DPI for line TG9 and at 0, 6, 8, 10 DPI for TG4. Initiation of symptom, disease index (DI) and lesion size were scored and measured according to Shiotani et al. [61] with slight modification using the Image J (Version 1.4.3.67) software. Unless otherwise specified, the sampled leaves were immediately immersed in liquid nitrogen, and stored at -80°C until use for experimental assay.

In another set of experiment, the leaves collected from new flushes of TG9 were washed with distilled water and divided into two groups, which were sprayed with either distilled water or 5 mM guazatine acetate (Sigma) supplemented with 2.5‰ of Tween-20. The treated leaves were cultured at 25°C under dark for 24 h before Xac inoculation as mentioned above. During Xac challenge, distilled water or guazatine acetate solution (5 mM) was added to the Petri dishes every day to maintain high humidity. The lesion areas were measured and DI was scored in the same way as mentioned above. In addition, the leaves sampled at 3 DPI were used for analysis of PAO activity, detection of HR and measurement of H_2_O_2_.

### In situ histochemical detection of H_2_O_2_

*In situ *accumulation of H_2_O_2 _at the inoculation site was detected by histochemical staining with 3, 3'-diaminobenzidine (DAB) based on a method of Shi et al. [46]. In addition, H_2_O_2 _level in the leaf discs adjacent to the inoculation site was determined at 2 DPI using a fluorescent probe H_2_DCF-DA [62]. In brief, the leaf discs collected from the vicinal regions with equivalent distance from the inoculated sites were cultured in Tris buffer (10 mM Tris, 50 mM KCl, pH 6.1), followed by incubation in a loading buffer (10 mM Tris, 50 mM KCl, pH 7.2) containing 50 μM of H_2_DCF-DA for 20 min in dark. The discs were then floated on fresh loading buffer to remove excessive dye, and observed under a Nikon 80i upright fluorescence microscope equipped with a mercury lamp (100 W) and a C-FL 25 mm Epi-Fluorescence Filter Block (MBE44500, B-2A) consisting of a 450-490 nm excitation filter, a 505 nm dichroic mirror and a 520 nm long-pass barrier filter. The approximate excitation and emission wavelengths of H_2_DCF-DA are 492-495 nm and 517-527 nm, while those of chlorophyll are 470-490 nm and >600 nm, respectively. Therefore, under our settings (excitation/emission = 450-490 nm/>520 nm), both the probe-derived fluorescence (green) and autofluorescence of chlorophyll (red) can be detected simultaneously. The CCD camera (Nikon D100) and the Nikon Capture 4 Camera Control software (version 4.1.0) were used to capture the images.

### Analysis of PAO, SOD, and CAT activity

Polyamine oxidase activity was determined in the leaf samples using a plant PAO assay kit (GMS50139.5, Genmed Scientifics Inc. USA) according to the supplier's instruction. About 1 g of leaf powder was extracted in 5 ml of lysis solution and the concentration of total protein was determined. Eight hundred μl of assay buffer were moved to a cuvette, where 50 μl sample (50 μg protein) and dilution solution were added to the sample and blank cuvette, respectively. After that, 100 μl of probe was added and the mixture was allowed to equilibrate for 2 min at 37°C. The reaction was initiated by adding 50 μl of the substrate, and the absorbance at 440 nm was continuously read for 5 min in a spectrophotometer (Varian Cary 50 Scan, Australia). The PAO activity was calculated according to the formula provided by the kit. SOD and CAT activities were measured using detection kits for SOD (A001, Nanjing Jiancheng Bioengineering Institute, China) and CAT (A007, Nanjing Jiancheng Bioengineering Institute, China), respectively, as descried by the supplier's instruction.

### Expression analysis of defense-related genes via quantitative real-time RT-PCR (qRT-PCR)

qRT-PCR was used to examine expression levels of defense-related genes in the transgenic plant and the WT before and after the Xac inoculation. Specific primers of the genes were designed using ABI Primer Express software version 2.0 based on the available sequences deposited in GenBank (Table 1), and specificity of the primers was further analyzed via primer-blast in NCBI database. The PCR amplification was performed using the Roche Lightcycler 480 Real-time PCR instrument and the SYBR Green Realtime PCR Master Mix (Toyobo, Japan) with the following programme: 95°C denaturation for 30 s, and then 45 cycles of denaturation at 95°C for 5 s, annealing at 54°C for 10 s, elongation at 72°C for 15 s. Each sample was amplified in tetraplicate.

### Statistical analysis

Xac inoculation of the WT and TG without PAO inhibitor treatment was repeated 5 times with 4 replicates in each time, and inoculation of the leaves treated with PAO inhibitor was performed twice. The presented data herein are mean values of a representative experiment, shown as the mean ± SE. The data were analyzed using SAS statistical software, and analysis of variance (ANOVA) was used to compare the statistical difference based on Fisher's Least Significant Difference (LSD) test, at significance level of *P *< 0.05 (*), *P *< 0.01 (**) and *P *< 0.001 (***).

## Authors' contributions

JHL conceived and designed experiments and CWC and XZF carried out the experiment. YW helped with the Xac inoculation. XZF drafted the manuscript, and JHL revised critically the manuscript and finalized it. TM provided the *MdSPDS1 *and helped to analyze the data. All of the authors have read and approved the final manuscript.
